# Microplastics in Lake Mead National Recreation Area, USA: Occurrence and biological uptake

**DOI:** 10.1371/journal.pone.0228896

**Published:** 2020-05-04

**Authors:** Austin K. Baldwin, Andrew R. Spanjer, Michael R. Rosen, Theresa Thom

**Affiliations:** 1 Idaho Water Science Center, U.S. Geological Survey, Boise, Idaho, United States of America; 2 Washington Water Science Center, U.S. Geological Survey, Tacoma, Washington, United States of America; 3 California Water Science Center, U.S. Geological Survey, Carson City, Nevada, United States of America; 4 Lake Mead National Recreation Area, U.S. National Park Service, Boulder City, Nevada, United States of America; Purdue University, UNITED STATES

## Abstract

Microplastics are an environmental contaminant of growing concern, but there is a lack of information about microplastic distribution, persistence, availability, and biological uptake in freshwater systems. This is especially true for large river systems like the Colorado River that spans multiple states through mostly rural and agricultural land use. This study characterized the quantity and morphology of microplastics in different environmental compartments in two large reservoirs along the Colorado River: Lakes Mead and Mohave, within Lake Mead National Recreation Area. To assess microplastic occurrence, surface water and surficial sediment were sampled at a total of nine locations. Sampling locations targeted different sub-basins with varying levels of anthropogenic impact. Las Vegas Wash, a tributary which delivers treated wastewater to Lake Mead, was also sampled. A sediment core (33 cm long, representing approximately 19 years) was extracted from Las Vegas Bay to assess changes in microplastic deposition over time. Striped bass (*Morone saxatilis*), common carp (*Cyprinus carpio*), quagga mussels (*Dreissena bugensis*), and Asian clams (*Corbicula fluminea*) were sampled at a subset of locations to assess biological uptake of microplastics. Microplastic concentrations were 0.44–9.7 particles/cubic meter at the water surface and 87.5–1,010 particles/kilogram dry weight (kg dw) at the sediment surface. Sediment core concentrations were 220–2,040 particles/kg dw, with no clear increasing or decreasing trend over time. Shellfish microplastic concentrations ranged from 2.7–105 particles/organism, and fish concentrations ranged from 0–19 particles/organism. Fibers were the most abundant particle type found in all sample types. Although sample numbers are small, microplastic concentrations appear to be higher in areas of greater anthropogenic impact. Results from this study improve our understanding of the occurrence and biological uptake of microplastics in Lake Mead National Recreation Area, and help fill existing knowledge gaps on microplastics in freshwater environments in the southwestern U.S.

## Introduction

Our understanding of microplastics in the environment has increased dramatically over the past decade. Defined as plastic particles < 5 mm in diameter, microplastics come from a wide variety of sources including fibers from textiles, preproduction pellets and powders, microbeads from personal care products, and breakdown of primary plastics such as bags, bottles, wrappers, packing foam, and car tires [1–5]. Microplastics reach aquatic environments via numerous and diverse pathways including littering, stormwater runoff, domestic and industrial wastewater, atmospheric deposition, and direct loss from buoys, boats, and other aquatic equipment [6–8]. Recent studies have highlighted the ubiquity of microplastics in virtually all compartments of the environment, from the ocean surface to its deepest trenches, in freshwater rivers and lakes, in precipitation, and in alpine snowpack [6,9–13]. Ingestion of microplastics has been observed across a wide range of aquatic organisms, from zooplankton to birds to whales [14–16]. However, biological risk assessment is complicated by the diversity of microplastic particle sizes, morphologies, and chemical compositions [17]. A growing body of evidence points to adverse biological effects, mostly at the sub-organismal level, but toxicological benchmarks have yet to be established, and organismal and community-level impacts remain largely unclear [18–21].

North American freshwater studies of microplastics have primarily focused on the Great Lakes region [6,9,22–24]. There is a general lack of information on microplastic occurrence and biological uptake in freshwater in the western portion of the continent, and especially the arid southwestern U.S. This investigation presents the first assessment of microplastic occurrence and biological uptake in two large reservoirs in the Mojave Desert, Lakes Mead and Mohave, located in Lake Mead National Recreation Area (LMNRA), a unit of the U.S. National Park Service. We report microplastic concentrations in water, surficial sediment, fish, and shellfish across a gradient of anthropogenic impacts to test the hypothesis that higher microplastic concentrations will be found in areas with higher anthropogenic impact. Additionally, we report microplastic concentrations from a sediment core representing ~19 years of deposition to test the hypothesis that rates of microplastic deposition have increased over time. Results from this study provide an initial baseline for microplastic occurrence and biological uptake in two large reservoirs along the Colorado River, in addition to an initial understanding of how human population density influences microplastic concentrations in a largely undeveloped watershed.

## Methods

### Study area

**Fig 1:**
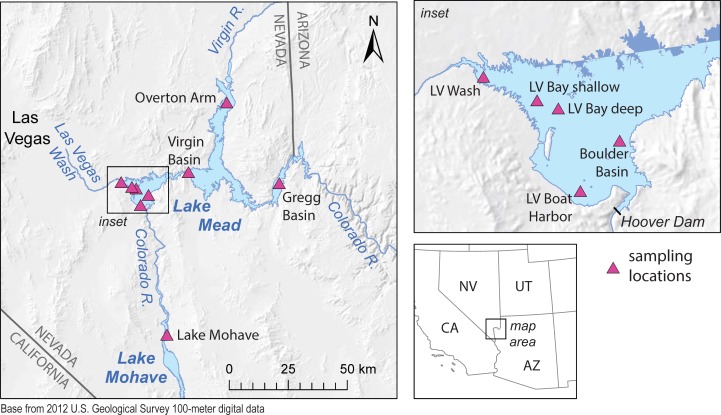
Sampling locations in Lake Mead and Lake Mohave. Lake boundaries are from the U.S. Geological Survey National Hydrography Dataset and are based on full-pool conditions, giving the false appearance that the Las Vegas Wash sampling location was in Lake Mead; at the time of sampling, the water level in Lake Mead was ~43 m below full pool, and the Las Vegas Wash sampling location was riverine. Hillshades are from U.S. Geological Survey 100-meter digital data. LV, Las Vegas.

Lakes Mead and Mohave are reservoirs along the Colorado River on the Arizona-Nevada border in the arid southwestern U.S. (Fig 1). Together these reservoirs cover 759 km^2^ [25] within the 6,070 square kilometer LMNRA. The reservoirs support a diverse population of benthic invertebrates, wintering bald eagles and other aquatic dependent birds, endangered fishes, and sportfish, which serve as an important food source for aquatic birds and are a major draw for park visitors. Lake Mead supports one of the world’s only self-sustaining populations of the critically endangered razorback sucker (*Xyrauchen texanus*) [25].

Lakes Mead and Mohave span a gradient of anthropogenic impacts. The Colorado River and its tributaries make up one of the major river basins of western North America. The basin upstream of Lake Mead includes portions of Wyoming, Colorado, Utah, New Mexico, Nevada, and Arizona. The Colorado River enters Lake Mead at Gregg Basin after flowing through Lake Powell and Glen Canyon dam in Glen Canyon National Recreation Area, and through Grand Canyon National Park. These are areas of sparse population but extremely high and increasing recreational use. For example, in 2018, Glen Canyon had 4.2 million visitors, and the Grand Canyon had 6.4 million visitors [26]. The Virgin and Muddy Rivers enter Lake Mead at Overton Arm. There are numerous small towns located along the Virgin and Muddy Rivers, but in general the basins are sparsely populated. The Virgin River flows through Utah, Arizona, and Nevada, and was designated Utah’s first wild and scenic river in 2009.

Annual visitation to the LMNRA was 7.5 million people in 2018 [27]. Visitation is heaviest in Boulder Basin and Las Vegas Bay in the western portion of Lake Mead, nearest the Las Vegas Metropolitan Area. Boulder Basin is home to the largest marina in the LMNRA, the Las Vegas Boat Harbor. The northern and easternmost portions of Lake Mead, Overton Arm and Gregg Basin, respectively, are farther from population centers and receive fewer visitors.

The majority of sediment entering Lake Mead comes from the Colorado River, but most dissolved or particle-bound contaminants entering Lake Mead come from Las Vegas Wash. The Las Vegas Wash is a tributary which delivers urban runoff, stormwater, and effluent from four wastewater treatment plants (719 million liters/day) in Las Vegas Valley to Las Vegas Bay [25]. Previous studies have shown that the relatively high contaminant loads from Las Vegas Wash have resulted in endocrine disruption of fish that primarily reside in Las Vegas Bay [28–34], although enhancements of the wastewater treatment plants since the early 2000s have reduced the overall chemical load [34,35]. However, none of these previous studies have examined the role of microplastics in the Lake Mead foodweb.

### Sample collection

Samples were collected in March 2017 and March 2018 (sediment core) at eight reservoir locations (seven in Lake Mead and one in Lake Mohave), and one tributary location (Las Vegas Wash; Fig 1, Table 1). The Las Vegas Wash sampling location appears to be in Lake Mead in Fig 1, but because of the low reservoir level was actually a riverine environment at the time of sampling. A total of 48 samples were collected: water and surficial sediment samples were collected at all locations; sediment cores, fish, and shellfish were collected at a subset of locations. Sample collection protocols were approved under National Park Service Research Permit LAKE-2017-SCI-0001.

**Table 1:** Sampling locations and types and numbers of samples collected.

Location name	Location prefix	Coordinates		Number of samples						
				Water	Sediment		Fish		Shellfish	
		Westing	Northing		Surficial sediment	Sediment core	Striped bass	Common carp	Asian clams	Quagga mussels
Las Vegas Boat Harbor	HAR	700667	3989780	1	1				2	
Las Vegas Bay Shallow	LVS	697116	3997438	1^a^	1^a^	4^b^	8	6	1	1 (11^c^)
Las Vegas Bay Deep	LVD	698925	3996756	1	1					
Boulder Basin	BOL	704121	3994026	1	1					
Virgin Basin	VIR	721071	4003667	1	1					
Overton Arm	OVA	737338	4033192	1^a^	1^a^		7			1 (10^c^)
Gregg Basin	GRG	759564	3998988	1	1					
Lake Mohave	MOH	711976	3934788	1	1					
Las Vegas Wash	LVW	692580	3999436	1	1					
TOTALS				9	9	4	15	6	3	2

Coordinates are in NAD 1983 UTM Zone 11N.

^a^ Replicate sample collected.

^b^ One core subsectioned into four samples.

^c^ Individuals were composited into a single sample for analysis.

#### Water samples

**Fig 2:**
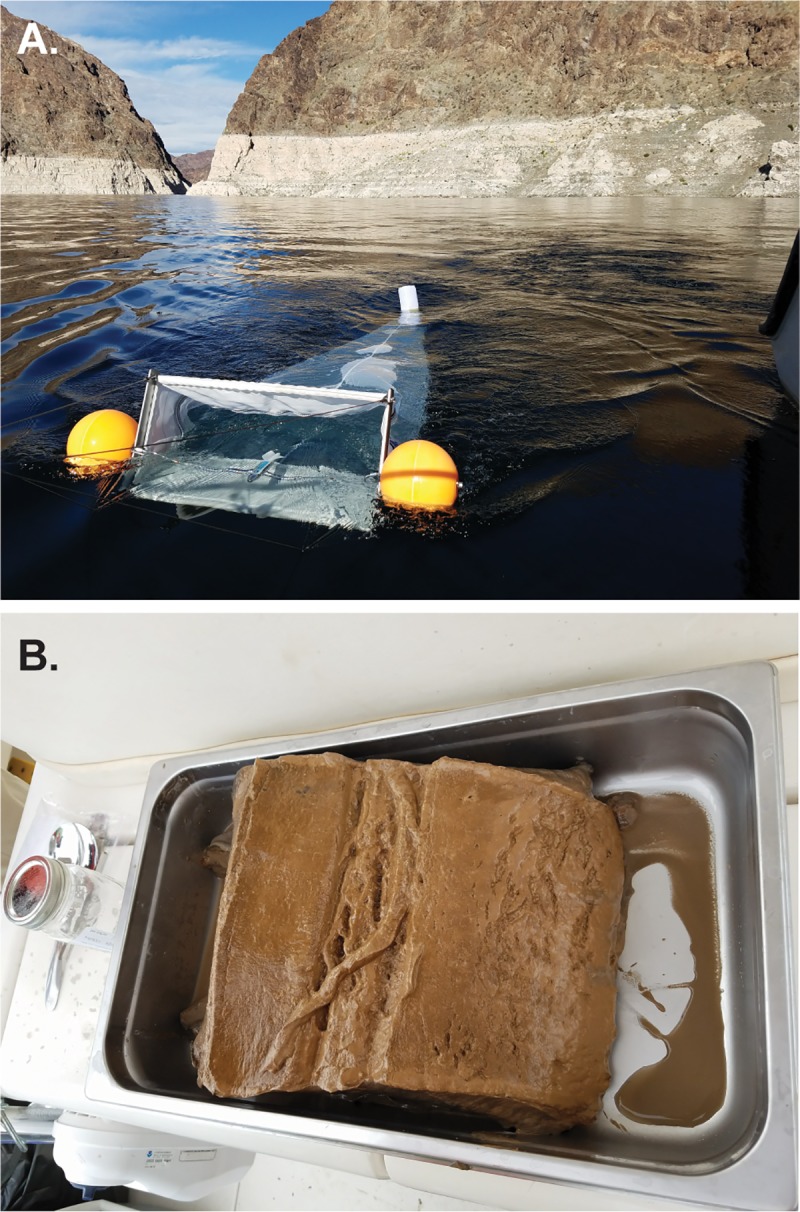
(A) Water sample collection net. (B) Sediment from Ponar sampler.

Water samples were collected once at each of the nine locations using methods and equipment similar to previous studies [6]. A 70 x 40 x 260 cm (width x height x length) microplastics net (HYDRO-BIOS) with 100 μm polyamide mesh was towed at the water surface for fifteen minutes (Fig 2A), except at Las Vegas Wash where the net was held at a fixed location (described below) and sampling was stopped after 5 minutes because the net began to clog. Floats on each side of the net’s frame kept the frame at a consistent depth through the duration of sample collection and across sites. The average submerged depth of the net was 22 ± 2.8 cm. For boat samples, the net was towed at 0.6–0.9 m/s alongside the boat beyond the bow wake using a fixed metal pole. At Las Vegas Wash, two people held the net in the stream and care was taken to keep the opening of the net upstream of where they were standing. A flow meter (General Oceanics, Inc, model 2030R) suspended in the mouth of the net was used to measure the average velocity of water entering the net. The total volume of water filtered (sampled) was calculated from the width and height of the submerged portion of the net, the sampling duration, and the average velocity. Total filtered volumes ranged from 10 to 122 cubic meters (m^3^).

Following sample collection, the net was suspended and sprayed with site water using a 70 pounds/inch^2^ wash down pump (Johnson Pumps model 10-13399-0311) to wash the sampled material down into the detachable polyamide mesh bucket at the bottom of the net. The net was sprayed primarily from the outside for ~2 minutes. The volume of site water used to wash the net was determined to equal ~19 L, which is equivalent to 0.02 to 0.2% of the sampled volume. Thus, any microplastic contribution to the sample from washing the net with site water was negligible. After washing the net, the sample was transferred from the mesh bucket to a glass jar using a squirt bottle and site water. The sample was preserved with isopropyl alcohol.

#### Surficial sediment samples

Surficial sediment was collected once at each of the nine locations using a standard Ponar sampler (6”W x 6”L Stainless Steel Ponar Grab sampler). Water depths at sediment sampling locations were 0.3 m at Las Vegas Wash and 3.6–99 m elsewhere. Water depth at Las Vegas Bay Shallow was 16 m, and at Las Vegas Bay Deep was 51 m. Ponar sediments were emptied into a stainless-steel pan and the sample was taken from the top ~3 cm using a stainless-steel spoon (the stratigraphy of the sediment remained generally intact during transfer to the pan; Fig 2B). At Las Vegas Wash, surficial sediment was collected from the top ~3 cm from depositional areas by wading and using a stainless-steel spoon. Approximately 400 mL of surficial sediment was collected at each location, composited into a glass jar and preserved with isopropyl alcohol. The Ponar, pan, and spoon were rinsed with site water between each location.

#### Sediment cores

A sediment core was collected at Las Vegas Bay Shallow using a gravity corer with polycarbonate barrel measuring 1.2 meters long and 6.8 cm inner diameter. The core penetrated to a depth of 33 cm. After collection, the core was capped and taken to shore for extrusion and sectioning into four 8–9 cm intervals. Core sections were collected and stored in glass jars at room temperature.

#### Fish

Fish were collected by boat electroshocking as part of routine fish population monitoring at two locations to assess potential differences in microplastic ingestion with differences in anthropogenic impact. The location considered to have relatively high anthropogenic impact was Las Vegas Bay, and the relatively low impact site was Overton Arm. Both benthic- and pelagic-feeding fish were targeted to assess potential differences in microplastic ingestion related to feeding strategy. Striped bass were chosen to represent pelagic feeders and common carp were chosen to represent benthic feeders. A total of 21 fish were collected: 8 striped bass and 6 common carp at Las Vegas Bay, and 7 striped bass at Overton Arm. No common carp were available at Overton Arm. Fish were euthanized by a sharp blow to the base of the skull. Fish lengths and weights are provided in S1 Table. The complete gastrointestinal tract (esophagus, stomach, and intestines) was removed, wrapped in aluminum foil, and stored frozen in a plastic bag until analysis.

#### Shellfish

Quagga mussels were first detected in Lake Mead in 2007 and have become abundant on all hard surfaces and in soft sediment [36]. Asian clams have been present in Lake Mead since at least the 1970s, but their introduction to Lake Mead is unknown [37]. Since the introduction of quagga musssels, Asian clam abundance has fallen and, therefore, quagga mussels were the target shellfish species for collection. As with fish, quagga mussels were collected at two locations, Las Vegas Bay (n = 11) and Overton Arm (n = 10), to assess potential differences related to adjacent land use. Quagga mussels were collected using a Ponar sampler (6”W x 6”L). Although not targeted, Asian clams were found in the surficial sediment samples at two locations (Las Vegas Boat Harbor and Las Vegas Bay) and were kept for analysis. Shellfish were euthanized by freezing, then were removed from their shells and stored frozen until analysis. Because of their small size, the quagga mussels from each site were composited for analysis, whereas the Asian clams were analyzed individually. Shellfish sizes are provided in S1 Table.

### Sample analysis

For sediment and water samples, a modified version of the National Oceanic and Atmospheric Administration's (NOAA) microplastic analysis method [38] was used to isolate and count microplastics. Briefly, for water, 1) whole samples were sieved into a single size class of 355 to 5,600 μm to remove small and large material; 2) organic non-plastic material was removed via a wet peroxide oxidation (WPO; Fenton’s reaction) digestion using 20 mL of 30% hydrogen peroxide and an iron catalase, additional hydrogen peroxide was frequently added to remove additional organic material; 3) remaining material was then sieved into two size classes, 355 to 1,000 μm and 1,000 to 5,600 μm; 4) each size class was density separated with lithium metatungstate (1.6 g/mL) and the less dense fraction retained on a sieve; lastly, 5) plastics and remaining “indigestible” material were transferred from the sieve onto a 47 mm gridded cellulose acetate filter paper using water and vacuum filtration and visually identified using a stereoscope capable of 40x magnification. The use of a 355 μm sieve size is a departure from the NOAA method, which used a 300 μm sieve size. As a result, microplastic concentrations reported in this study are likely slightly lower than they would be with a 300 μm sieve, which may be an important consideration for comparison with other studies.

Identified plastics were categorized based on their morphology as fragments, fibers, foams, beads/pellets, and films [9]. Both color and counts were recorded. Sediment samples were processed with the same methods as water samples with three additional initial steps: 1) sediments were dried at 90°C to obtain a sediment dry weight; 2) 500 mL of potassium metaphosphate solution (5.5 g/L) was stirred with dry sediment to disaggregate sediment; and 3) an initial density separation was performed in lithium metatungstate to separate plastics and organic material from the sediment. Only material with a density of less than 1.6 g/mL was retained for subsequent digestion and size separation.

Tissue samples were also processed via visual counting with stereoscope and classification of their morphology and color recorded. For fish, processed tissues included the complete gastrointestinal tract; for mussels, the whole organism was processed, excluding the shell. To isolate plastics, mussel and fish tissue were placed into a solution of 30% potassium hydroxide (KOH) and allowed to digest for at least 24 hours [39]. Additional time was often needed to digest depending on the amount of tissue. For mussels, digestate was first filtered with a 125 μm sieve to remove KOH and then transferred to a 47 mm gridded cellulose acetate filter paper using water and vaccum filtration for plastic identification and counting. Both striped bass and common carp tissue samples had a large amount of shell and rock material in their GI tract requiring removal. After removal of KOH with a 125 μm sieve, fish digestate was treated with a dilute solution of 5% hydrochloric acid to dissolve shells followed by a density separation in 1.6 g/mL LMT solution to isolate plastics from rocks and transferred to a 47 mm gridded cellulose acetate filter paper for plastic identification and counting. KOH digestion was used due to its efficacy at dissolving organic tissues and the fact that most plastics are resistant to degradation from KOH [39]. The size of microplastics counted in mussels and fish was larger than 125 μm and no size separation was completed due to the relatively small amount of plastic in tissues and no large macroplastics (>5.6 mm) present.

### Data analysis

Plastic particle concentrations are reported in particles/m^3^ in water samples, particles per kilogram dry weight (kg dw) in sediment samples, and particles per organism in fish and shellfish. Because quagga mussels were composited by site, results are averages across 10–11 organisms.

Comparisons of fish lengths and microplastic concentrations between different locations and species were done by two-sample unpaired Wilcoxon Rank Sum tests with a significance level (*p* value) of 0.05 using the wilcox.test function in R [40]. Normality of fish lengths and fish microplastic concentrations was assessed using the Shapiro-Wilk’s test in R [40]. Relations between fish length and microplastic concentration were assessed by site and species using Spearman correlation with a *p* value of 0.05. Spearman correlations were calculated in R using the rcorr function in the Hmisc package [41].

All sample results are available online in the USGS ScienceBase Catalog [42].

### Quality assurance and quality control

Quality assurance and quality control included field replicates, field blanks, and laboratory blanks. Field replicates of water and sediment samples were collected at two locations. The relative percent difference (RPD) in total microplastic concentration between replicate pairs in water samples was 9.5% (Overton Arm) and 24.9% (Las Vegas Bay shallow), and in sediment samples was 57.4% (Overton Arm) and 25.1% (Las Vegas Bay shallow).

Field blanks consisted of sample jar blanks and a field equipment blank. The purpose of the sample jar blanks was to assess potential contamination from the glass sampling jars and from the atmosphere while the sampling jars were open. The sample jar blanks were collected by leaving a clean, unrinsed sample jar open to the outdoor atmosphere for approximately five minutes. Four sample jar blanks were collected for this and concurrent studies, with an average ± standard deviation (SD) of 3.5 ± 5.7 microplastic particles per jar (range 0–12; S2 Table). The contribution of fibers from the atmosphere versus what was in the jar to begin with (i.e., from the factory) is not known. Based on these results we recommend future studies pre-rinse sample jars to minimize this potential contamination source. The purpose of the field equipment blank was to assess the potential contamination from using site water to wash the nets, as well as potential contamination from the net, plastic components in the pump, and carryover from the previously-collected sample. The field equipment blank was collected by spraying site water from Las Vegas Bay through the net bucket for 5 minutes (greater than twice the time and volume typically used to wash a net), then washing any collected material from the net bucket into a sampling jar. Two plastic fibers were reported in the field equipment blank (S2 Table). The contribution of fibers from the net versus the atmosphere versus the jar is not known. The small number of microplastic particles in the field equipment blank indicate that the net and the pump were not major sources of contamination, and that the net was being adequately rinsed between sites.

Laboratory blanks consisted of process blanks and equipment blanks. Laboratory process blanks were collected to determine the potential contamination of samples from atmospheric exposure at the laboratory. Fifty laboratory process blanks were collected concurrent with the analysis of regular samples by setting an open petri dish alongside the analyst for the duration of sample processing. The number of microplastic particles in laboratory process blanks ranged from 0 to 17, with a median of 1.0 and an average ± SD of 2.6 ± 3.5 (S2 Table). Laboratory equipment blanks were collected by analyzing samples of deionized water to confirm clean handling of samples, processing solutions, and equipment, and potential crossover contamination between samples. Seven laboratory equipment blanks were run. The number of microplastic particles in laboratory equipment blanks ranged from 2 to 6, with a median of 3 and an average ± SD of 3.6 ± 1.4 (S2 Table).

In summary, results from field and laboratory blanks indicate a combined contamination estimate of 11.7 microplastic particles per sample, on average (median 7; range 4–37). For context, environmental water and sediment samples averaged 110 and 114 total particles, respectively (water samples ranged from 38 to 243 particles, sediment samples ranged from 28 to 425 particles). Environmental results were not blank-subtracted based on laboratory or field blank results [17,43–45]. Rather, blank results provide the range of potential contamination during field and laboratory protocols.

## Results

### Water

**Fig 3:**
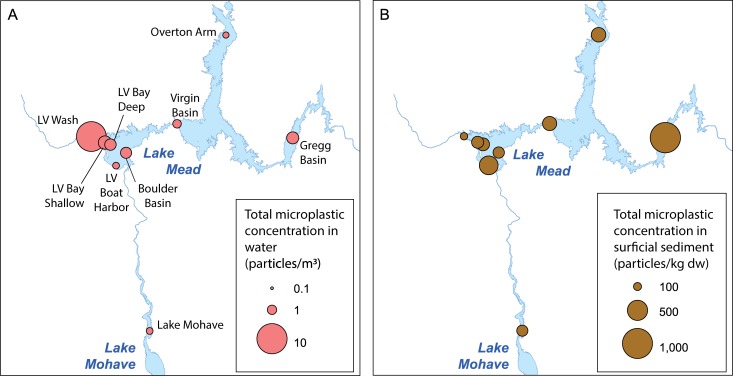
Total microplastic concentrations in (A) water and (B) surficial sediment samples, Lake Mead and Lake Mohave, 2017. Las Vegas Wash (LV Wash) is a stream sample, all others are lake samples. Lake boundaries are from the U.S. Geological Survey National Hydrography Dataset. dw, dry weight.

**Fig 4:**
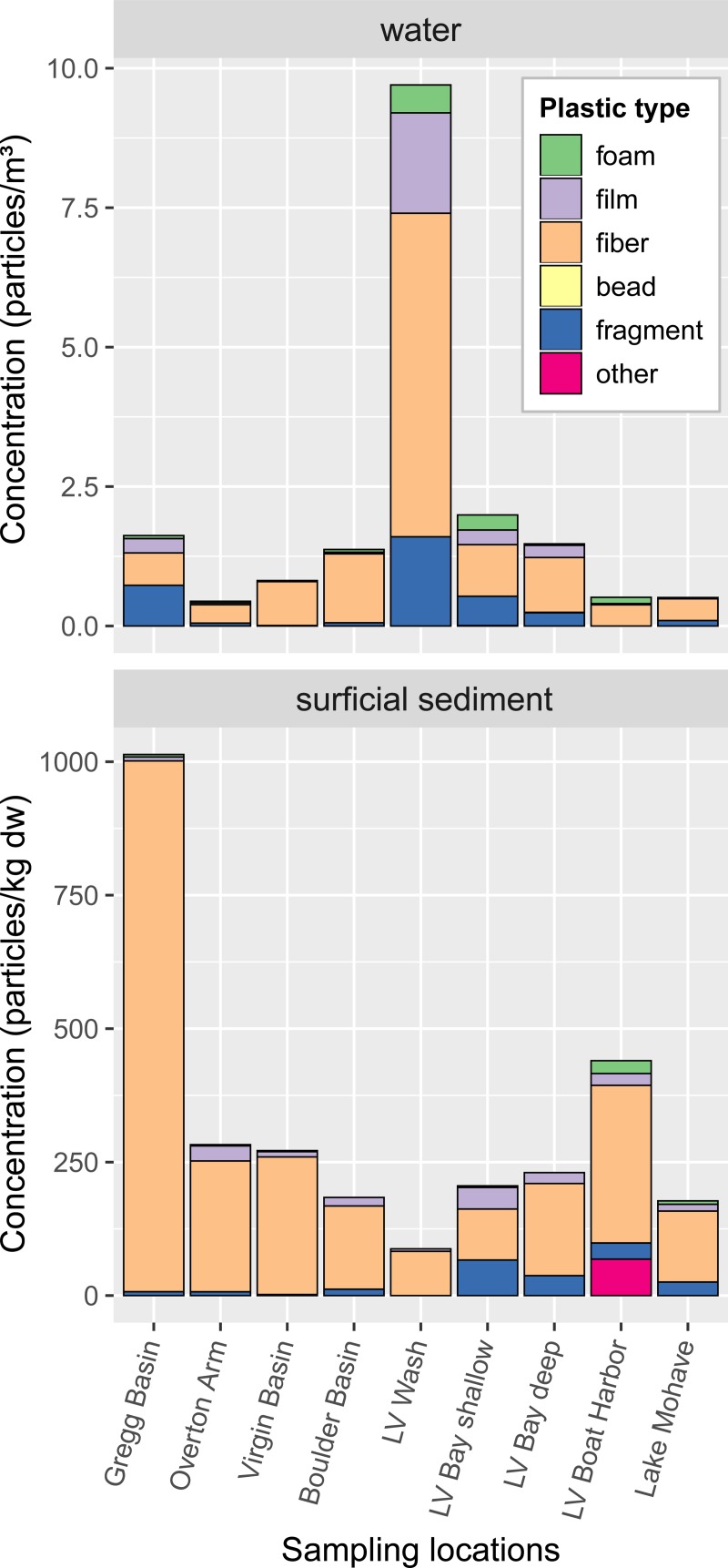
Concentrations of different microplastic types in water and surficial sediment samples, Lake Mead and Lake Mohave, 2017. dw, dry weight; LV, Las Vegas.

Microplastic concentrations in water samples were 0.44–1.99 particles/m^3^, except at Las Vegas Wash, where the concentration was 9.7 particles/m^3^ (Figs 3A and 4). Fibers comprised 68.9% of the microplastic particles in water samples, on average, followed by fragments (15.6%), films (8.9%), foams (6.5%), and beads/pellets (0.1%). Non-fibrous particles (namely fragments, films, and foams) were primarily found in samples from Las Vegas Wash, Las Vegas Bay, and Gregg Basin. Most sampled particles (73.1%) were in the 355–1,000 μm size range, while 26.5% were in the 1,000–5,600 μm size range and 0.4% were >5,600 μm. The predominant colors of microplastic particles in water samples were clear (33.4% average), white (18.7%), black (17.1%), blue (14.7%), and red (6.7%).

### Surficial sediment

Microplastic concentrations in surficial sediment samples were highest at Gregg Basin (1,010 particles/kg dw), followed by Las Vegas Boat Harbor (440 particles/kg dw; Figs 3B and 4). Elsewhere, surficial sediment concentrations were 87.5–283 particles/kg dw. On average, 80.3% of particles in surficial sediment samples were fibers, followed by fragments (8.9%), films (7.7%), foams (1.4%), and “other” particles (1.7%). Fragments were primarily found in samples from Las Vegas Bay, Las Vegas Boat Harbor, and Lake Mohave, whereas films were found at low concentrations at all sites. The “other” particles were only found at the Las Vegas Boat Harbor, where they made up 15.5% of the sample. These were black, rubbery particles that were suspected of being tire wear particles, though that was not confirmed. Tires are used extensively in the Las Vegas Boat Harbor as boat bumpers and to form a large, approximately 1.5 km-long floating breakwall around the harbor.

Most sampled particles (63.7%) were in the 355–1,000 μm size range, while 36.3% were in the 1,000–5,600 μm size range. The predominant colors of microplastic particles in surficial sediment samples were clear (37.8% average), black (26.2%), blue (24.3%), and red (6.5%).

### Las Vegas Bay sediment core

**Fig 5:**
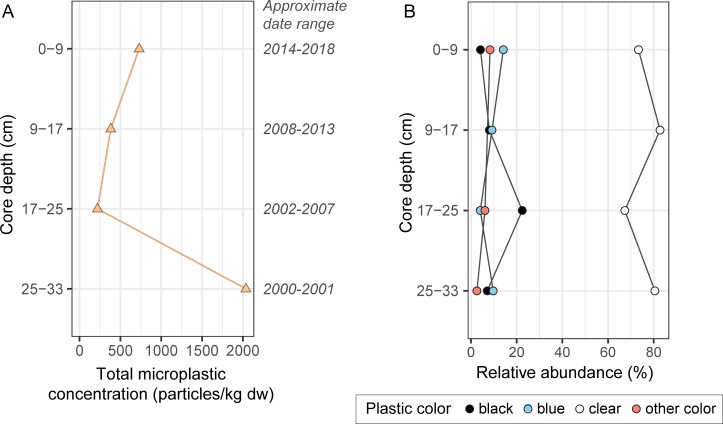
Concentrations of different microplastic types (A) and relative abundance of microplastic colors (B) in sediment core samples from Las Vegas Bay, Lake Mead, 2018. dw, dry weight.

Based on a previously published sediment mass accumulation rate of ~1.0 g/cm^2^/year in Las Vegas Bay [46], the 33 cm-long sediment core represented approximately 19 years of deposition (2000–2018). Concentrations of microplastics in the four sectioned samples of the core ranged from 220 to 2,040 particles/kg dw, with no consistent trend with depth/time, although the highest concentration was in the deepest sample (Fig 5A). Virtually all (99.3%) of the microplastic particles in core samples were fibers. Most sampled particles (72.0%) were in the 355–1,000 μm size range, while 28.0% were in the 1,000–5,600 μm size range. On average, 77.3% were clear, 10.2% were blue, 8.2% were black, and 4.2% were red and other colors. The predominance of clear particles was relatively consistent with depth, varying from 67.3% to 82.8% (Fig 5B).

### Fish

**Fig 6:**
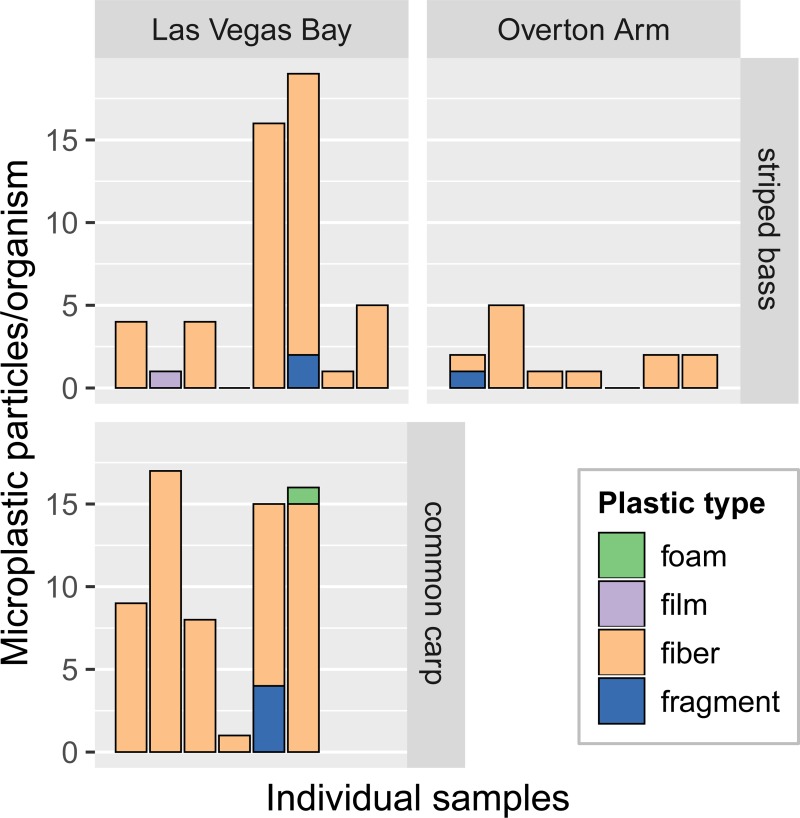
Concentrations of different microplastic types in striped bass and common carp, Lake Mead, 2017. Common carp were not sampled at Overton Arm. LV, Las Vegas.

Microplastic concentrations in striped bass across both sampling locations ranged from 0–19 particles/organism (mean 4.2, median 2.0; Fig 6). Striped bass from Las Vegas Bay (mean 6.3, median 4.0 particles/organism) had higher (not statistically significant) microplastic concentrations per fish than striped bass from Overton Arm (mean 1.9, median 2.0 particles/organism). There was no significant difference in fish lengths between the two locations (*p* = 0.41). Common carp at Las Vegas Bay (mean 11.0, median 12.0 particles/organism) had higher microplastic concentrations per fish than striped bass at the same location (not statistically significant). Although the common carp were significantly larger than the striped bass at Las Vegas Bay, species length was not correlated with microplastic concentration. Fibers made up on average 90.7% of all microplastic particles in fish samples. The predominant colors of microplastic particles in fish samples were clear (37.2% on average), blue (29.5%), black (17.1%), and red (9.3%).

### Shellfish

**Fig 7:**
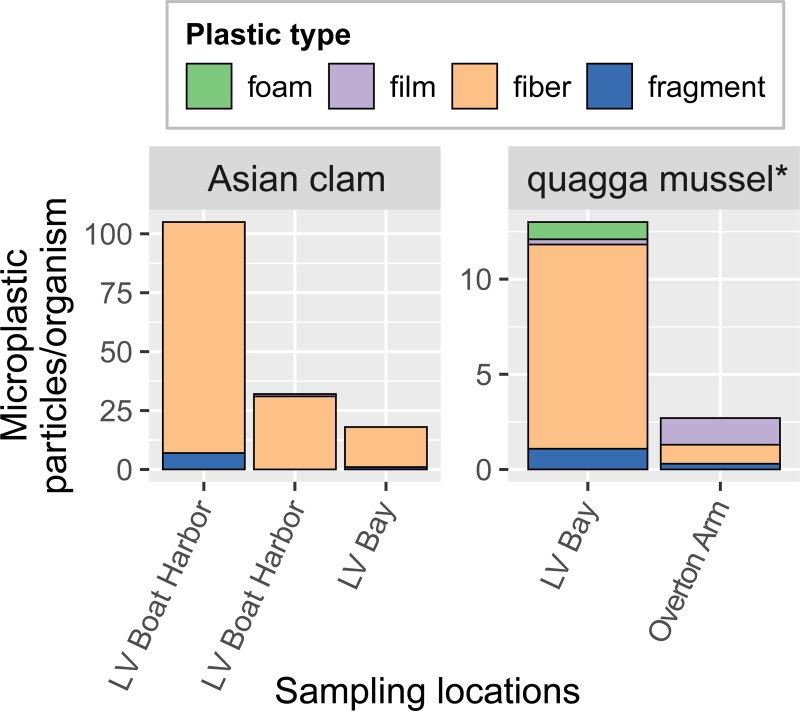
Concentrations of different microplastic types in Asian clams and quagga mussels, Lake Mead, 2017. *Average from 10–11 individuals, composited.

Concentrations of microplastics in Asian clams ranged from 18–105 particles/organism (mean 51.7 particles/organism, n = 3; Fig 7). In quagga mussels, microplastic concentrations were higher at Las Vegas Bay (13.0 particles/organism averaged over 11 composited individuals) than at Overton Arm (2.7 particles/organism averaged over 10 composited individuals), despite the Overton Arm quagga mussels being approximately double in their size (shell long axis length of 0.6–1.4 cm at Las Vegas Bay versus 1.8–2.2 cm at Overton Arm; S1 Table).

The relative abundances of particle types in shellfish were similar to those in surficial sediment: on average, fibers made up 80.9% of particles, followed by films (11.4%), fragments (6.3%), and foams (1.4%)). The predominant colors of microplastic particles in shellfish samples were clear (42.5%), blue (23.1%), black (17.8%), and red (4.9%).

## Discussion

Results from this study show that microplastics were present in water and sediment throughout the study area and that ingestion by aquatic organisms was common. Microplastic concentrations (including concentrations in biota) appeared to be higher at locations with greater direct anthropogenic use and input (e.g., Las Vegas Wash, Las Vegas Bay, Las Vegas Boat Harbor), similar to results from studies looking at dissolved and particulate endocrine disrupting compounds in water, sediment, and fish [25,32–34,46,47]. An exception to this was surficial sediment from Las Vegas Wash, which had the lowest concentration among the sampled locations. We hypothesize that the flow velocity and turbulence at the Las Vegas Wash location kept most microplastic particles in suspension, thereby minimizing deposition. Another exception was Gregg Basin, a remote arm of Lake Mead far from Las Vegas or other large population centers, which had the highest microplastic concentration in surficial sediment, as well as high relative abundances of fragments and films in water. Much of the Colorado River Basin upstream of Gregg Basin is undeveloped, but upstream areas such as Grand Canyon National Park and Glen Canyon National Recreation Area receive heavy visitation, including water-based recreationists. Gregg Basin is where the flow of the Colorado River slows as it enters Lake Mead, so it is likely that any plastic particles in suspension will fall out and accumulate here as the river water slows and the depth of the water column deepens. We would expect a similar process to occur in Las Vegas Bay, where Las Vegas Wash enters the lake and velocities slow. Surficial sediment concentrations were comparatively low in Las Vegas Bay, however, perhaps simply due to choice of sampling locations.

Across all sample types, the most common color of microplastic particles was clear, making up 33.4–42.5% (on average) of particles in water, surficial sediment, fish, and shellfish, and 77.3% of particles in the sediment core. This abundance of clear particles may be attributed in part to the loss of color during environmental degradation and (or) bleaching during the WPO digestion of the sample in the laboratory [45]. Blue particles were also common across sample types, making up 10.2 to 29.5% of particles, on average. The source of blue particles is unclear but their prevalence has been noted by studies worldwide [44,45,48,49]. White particles were fairly common in the water samples (18%) but were rare or absent in other compartments. This difference may be because of polymer type (e.g., white particles tend to be a certain polymer type which is buoyant and therefore remains at the water surface), or it may be that in sediment and biota the once-white particles have degraded to clear.

As with other studies of microplastics in freshwater [6,50–52], in this study fibers were the most abundant microplastic type in all environmental compartments. Whereas fragments, films, and foams were more commonly found at locations with greater anthropogenic impact, fibers were ubiquitous across all locations. The widespread presence of fibers across the study area, including at relatively remote locations (e.g., Overton Arm, Virgin Basin), suggests a diffuse source. Increasingly, the atmosphere is being investigated as an important pathway for microplastic fiber transport to and deposition in remote locations. Recent studies in both urban areas and remote mountain environments have documented atmospheric deposition of microplastic fibers and other particle types (e.g., fragments) [8,11,49,53]. The atmospheric contribution of microplastics to Lakes Mead and Mohave is unclear but may be significant, particularly as the area is downwind of Las Vegas and other large metropolitan areas in California.

There is clear evidence of microplastics entering the food chain. The biological component of this study focused on a limited number of fish and shellfish species, but microplastic ingestion by other organisms is also likely. This includes other fish species, such as the endangered razorback sucker, as well as piscivorous birds such as osprey and bald eagles. Results here are conservative due to the relatively large size fraction of microplastics identified. Given the propensity for microplastic abundance to increase with smaller size fractions [54], it is likely that the number of ingested micro- and nanoplastics also increases with smaller size fractions (<150 μm). The biological effects of microplastic ingestion in the study area are unknown because toxicological benchmarks have yet to be established.

### Comparisons to previous studies

Comparison of results between microplastic studies is complicated by a lack of standardized field and laboratory methods [55–57]. In this section, comparisons to other studies are focused on freshwater studies which used methods similar to the current study. Where possible, we also compare results to those from the St. Croix National Scenic Riverway (hereafter St. Croix) and the Mississippi National River and Recreation Area (hereafter Mississippi) in Wisconsin and Minnesota (USA) [58]. The St. Croix and Mississippi are riverine rather than reservoir environments, but are relevant because they are the only other U.S. National Park Service waterbodies which have been sampled for microplastics using similar methods. More comprehensive reviews of microplastics in freshwater environments are available elsewhere [13,55,56,59].

Concentrations of microplastics in water samples in the current study (0.44–9.70 particles/m^3^) are somewhat lower than a reported value for Brownlee Reservoir, another large reservoir in the western U.S. (13.7 particles/m^3^; Snake River, Idaho/Oregon) [52]. Concentrations in water samples from the St. Croix and Mississippi Rivers (0.80–4.84 particles/m^3^) [58], and from the much more urban Milwaukee Harbor and nearshore Lake Michigan (Wisconsin, USA; 0.21–5.23 particles/m^3^) [60], were similar to those in the current study. However, the St. Croix/Mississippi and Milwaukee Harbor/Lake Michigan studies used a larger mesh size (333 μm) than the current study, potentially biasing their concentrations low.

Concentrations in surficial sediment samples in the current study (87.5–1,010 particles/kg dw) are similar to those reported in the Milwaukee Harbor and nearshore Lake Michigan (Wisconsin, USA; 39.5–319 particles/kg dw) [60]; nearshore Lake Ontario (Canada; 40–4,270 particles/kg dw) [23]; and an urban lake in London (England; 539 particles/kg dw) [61]. The overlap in sediment microplastic concentrations between Lake Mead NRA and these more urbanized locations may point to a dispersed pathway such as atmospheric deposition of microplastic particles on the landscape [8,11,53].

A review of microplastic ingestion by wild freshwater fish around the world found that 74% of studies (n = 23) reported mean concentrations of fewer than 3 particles/organism, and only 13% reported mean concentrations greater than 6 particles/organism (maximum average concentration 19.2 particles/organism) [62]. Based on these ranges, concentrations in fish in the current study are typical at Overton Arm (average 1.9 particles/organism in striped bass), but are somewhat high at Las Vegas Bay (average 6.3 particles/organism in striped bass, 11.0 particles/organism in common carp). However, this comparison is complicated by differences in fish species, sizes, trophic levels, and feeding habits among the different studies. In smallmouth bass from the St. Croix and Mississippi Rivers, concentrations were somewhat higher than the current study, averaging 17.5 particles/organism (range 1–111 particles/organism) [58].

Studies of microplastics in wild quagga mussels are lacking, but a study of Asian clams at 21 lake, river, and estuary locations in the Middle-Lower Yangtze River Basin (China) reported averages of 0.4–5.0 particles/organism [45]. Asian clam concentrations in the current study were considerably higher, averaging 51.7 particles/organism (range 18–105). However, the current study included only three Asian clams, all collected from areas of relatively high anthropogenic impact, and thus not likely representative of Lake Mead NRA in general. Concentrations in three-ridge mussels from the St. Croix and Mississippi Rivers averaged 6.7 particles/organism (range 1–18), similar to concentrations in quagga mussels in the current study (average of 2.7–13.0 particles/organism).

### Constraints on interpretations

Water and sediment were sampled only once at each location, and fish and shellfish sample sizes were also relatively small (especially those of Asian clams, which were not targeted but sampled opportunistically). RPDs of water and sediment sample replicates show considerable variability, highlighting the constraints on interpretations based on small sample numbers. Further study is needed to better understand the spatial and temporal variability in microplastic concentrations in LMNRA.

In addition to small sample sizes, another constraint on our interpretations is the sole reliance on visual microscopy for detection/identification of microplastics in samples. Studies have shown that microplastic identification using visual microscopy is susceptible to false-positives due to the difficulty in deciphering between plastic and natural materials, especially at sizes <100 μm [63,64]. As a result, spectroscopic methods such as Raman micro-spectroscopy or Fourier Transform Infrared Spectroscopy (FTIR) are increasingly being used, typically on a subset of particles, to confirm determinations made by visual microscopy. In a comparison of visual microscopy to Raman micro-spectroscopy, Lenz et al. [63] found that the false-positive rate using visual microscopy decreased with increasing particle sizes. For that study, at sizes <50 μm, 63% of particles that had been visually identified as plastic were confirmed using spectroscopic methods, but the confirmation rate increased to 83% for particles >100 μm. A study comparing visual microscopy to FTIR reported that fibers were overestimated using visual microscopy but that fragments were *underestimated*, and that total microplastic particle counts were actually *lower* using visual microscopy compared to FTIR [65]. Although spectroscopic confirmation was not used in the current study, we considered only particles >355 μm in sediments and water and >150 μm in tissues to minimize false-positives, despite water samples having been collected using a 100 μm net. Based on Lenz et al. (2015), this conservative approach should result in a relatively low rate of false positives, perhaps in the range of 15–20%.

Results may be biased low for cosmetic microbeads. A limited number of polymers are susceptible to degradation from the temperatures reached during processing (e.g., WPO and sediment drying). Specifically cosmetic microbeads that have non-plastic wax components [66] are likely to be removed during processing. WPO and temperatures up to 100°C have not been found to alter other common polymers (e.g. polyethylene, polystyrene, or nylon) [66].

## Conclusion

This study is a first look at microplastic occurrence in LMNRA, and helps fill existing knowledge gaps on microplastics in freshwater environments in the southwestern U.S. The results presented here provide a basic understanding of microplastic occurrence in different environmental compartments in Lakes Mead and Mohave, and they provide insights into sources, transport, and fate across a gradient of anthropogenic impacts. Further study is needed to better understand these processes. Although sample sizes are limited, results indicate greater uptake of microplastics by fish and shellfish in areas with greater anthropogenic impacts. The ecosystem effects of the observed microplastic pollution remain unclear, but will be increasingly important as the human population living and recreating in the Colorado River Basin continues to grow.

## Supporting information

S1 Table Locations and sizes of fish and shellfish samples. NA, not available (was not weighed because of size).
(XLSX)

S2 Table Summary of results from laboratory process and equipment blank samples. (XLSX)
